# Influence of ageing on human body blood flow and heat transfer: A detailed computational modelling study

**DOI:** 10.1002/cnm.3120

**Published:** 2018-07-23

**Authors:** Alberto Coccarelli, Hayder M. Hasan, Jason Carson, Dimitris Parthimos, Perumal Nithiarasu

**Affiliations:** ^1^ Biomedical Engineering Group, Zienkiewicz Centre for Computational Engineering, College of Engineering Swansea University UK; ^2^ Division of Cancer and Genetics, School of Medicine Cardiff University UK

**Keywords:** ageing, bioheat transfer, body thermal energy balance, systemic circulation, thermoregulation

## Abstract

Ageing plays a fundamental role in arterial blood transport and heat transfer within a human body. The aim of this work is to provide a comprehensive methodology, based on biomechanical considerations, for modelling arterial flow and energy exchange mechanisms in the body accounting for age‐induced changes. The study outlines a framework for age‐related modifications within several interlinked subsystems, which include arterial stiffening, heart contractility variations, tissue volume and property changes, and thermoregulatory system deterioration. Some of the proposed age‐dependent governing equations are directly extrapolated from experimental data sets. The computational framework is demonstrated through numerical experiments, which show the impact of such age‐related changes on arterial blood pressure, local temperature distribution, and global body thermal response. The proposed numerical experiments show that the age‐related changes in arterial convection do not significantly affect the tissue temperature distribution. Results also highlight age‐related effects on the sweating mechanism, which lead to a significant reduction in heat dissipation and a subsequent rise in skin and core temperatures.

## 1 INTRODUCTION

For the elderly human population, a sudden environmental change represents a serious threat that can put lives at risk and thus requires taking immediate and appropriate actions.^1^ This is due to the fact that ageing lowers thermal resistance and leads to a less efficient thermoregulatory system. Ageing is a process that can be defined as an intrinsic deterioration of the homeostatic capabilities of an organism, leading to a constantly increasing risk of death.^2^ The accumulation of defective mitochondria represents one of the most accredited factors causing ageing.^3^, ^4^, ^5^, ^6^ This is because the free radicals produced in oxidation can damage several biologically relevant components such as mitochondrial DNA, lipids, and proteins. Mitochondrial DNA mutations may drastically reduce cellular survival, because this DNA produces enzymes necessary for the oxidative phosphorylation. Biological and biochemical mechanisms, such as gene regulation and signal transduction mutagenesis, are coupled to biomechanical ones, giving rise to physiological ageing.

At tissue level, such irreversible cellular degradation leads to changes in organ functionalities, tissue physical properties, and volumes. Ageing effects on the human body may be amplified by the lifestyle and the environmental conditions that a subject is exposed to. Due to a less efficient immune system, elderly individuals are more vulnerable to pathogens than young adults. Furthermore, ageing also affects the capacity of the body to react to changes of either internal or external conditions, such as physical workload, food digestion, and thermal stress exposure. The thermal energy balance of the body depends on a range of physiological and anatomical components such as cardiac function, tissue volumes, thermal properties, and metabolic production and may be extremely sensitive to any variation of these factors. Elderly people generally present significant alterations of such components with respect to the younger individuals, which may involve markedly different thermal responses for the same external conditions.

The cardiovascular system undergoes various changes with age (*ξ*), leading to a significant increase in pressure amplitude along the arterial tree. The pulsating nature of arterial blood load triggers fatigue and fracture processes of elastin lamellae in large elastic arteries, causing progressive vessel wall stiffening and a consequent dilation with age. Such decrease in elasticity is associated to an increase in pulse wave velocity and thus for a stiffer arterial network, reflected waves return to the heart earlier. This reduction in compliance is especially valid for large elastic arteries.^7^, ^8^ The study by Segers et al^9^ also highlighted the modulatory role of ageing especially in the aorta. Although the link between ageing and cardiac function adaptation needs further elucidations, it is widely accepted that the cardiac compensation of such extra load occurs without a significant increase in ventricular wall stress but with a left ventricular wall thickening, which can be associated with a form of concentric hypertrophy.^10^ This, with age, leads to an aortic systolic pressure rise and a diastolic pressure fall. For a more exhaustive description of vascular changes due to ageing, see other studies.^11^, ^12^, ^13^ It is worth noting that these changes in the arterial flow may lead to a different heat exchange by convection with the surrounding tissues.

Ageing also has profound effects on the functional and structural properties of tissues. Jackson and Pollock^14^ provided a key contribution to the topic, by formulating a set of generalised equations for calculating body density with age. However, for modelling purposes, including specific tissue components allows to characterise more accurately ageing effects. Body tissues are subjected to gradual age‐related changes in volume. Although these volume variations are generally non‐linear as a function of ageing, the lack of experimental data generally makes it necessary to describe such relationships via linear regression techniques. With advancing age, the muscle mass is significantly reduced, along with a decrease in elasticity, strength, and functionality. Sarcopenia is defined as a progressive decline in skeletal muscle mass primarily due to ageing, but it may have other causes such as muscle disuse, inadequate nutrition, cachexia, and neurodegenerative diseases.^15^ Kuczmarski et al^16^ obtained some descriptive anthropometric data for elderly people that show that the decline in body mass index parallels the progressive weight reduction. Older people (*ξ* > 50 years) generally present subcutaneous fat reduction, which is commonly compensated by a slight increase in visceral fat. In the study by Bemben et al,^17^ it was shown how the percentage of fat and fat‐free mass in males varies between 20 and 74 years old. Across it's lifespan, bone tissue microstructure is subjected to several changes, some of which may lead to disease states, such as osteoporosis. As reported in the study by Emaus et al,^18^ bone density variations with age depend strongly on the gender. Both cortical and cancellous components contribute to differences in age‐related changes in bone tissue.^19^, ^20^ Chen et al.^21^ reported, for both genders, the density variation with age of a mid‐thoracic vertebral trabecular bone. It is reported that such density decline occurring for the trabecular component is much higher than the decrease occurring for the global bone. Ageing also affects the basal metabolic rate (BMR), which defines the energy requested by the body for guaranteeing homoeostasis at rest, under thermoneutrality conditions and fasting. As reported in the study by Ruggiero et al,^22^ this rate tends to decline at a pace that accelerates in older age. From a case study of 300 healthy men, Poehlman et al^23^ extracted a quadratic relationship between the resting metabolic rate (RMR) and age. Novieto^24^ drew inferences from data sets of 2 different studies (Oxford BMR and FAO/WHO/UNU BMR) for 2 different weight categories (65 and 75 kg).^25^ For the 75‐kg group, both studies showed a similar decrease in BMR with age at almost constant rate ≈ −0.38 W/y. However, these reduction rates extrapolated are only indicative, as such approaches do not account for age‐related body weight changes. It is indeed widely accepted that body weight increases with age, reaching a plateau during middle age. Thereafter, a gradual reduction occurs, which is inline with tissue thinning.

For healthy older people, the body core temperature under thermoneutral conditions does not significantly differ from the one in younger people. Kenney and Munce^26^ reported that, with respect to young adults, elderly people under heat stress conditions typically respond with attenuated sweating and decreased vasodilation. This is accompanied by reduced cardiac outputs (COs) and smaller redistributions of blood flow from the splanchnic and renal circulations. Ageing also affects the response during cold stress conditions, involving a reduced peripheral vasoconstriction and shivering energy production.

The difference in thermal response between different age groups becomes obviously more evident when the environmental exposure is extreme, such as in the case of heat waves or hypothermic conditions. Due to their limited sensory perception, however, elderly people are also at risk when they are exposed to mild environmental conditions for long times. When the subject is exposed to a hot stress environment, the body temperature increase is contrasted by the action of the thermoregulatory system, which strives to keep the core temperature within the thermoneutrality range (approximately 36.8°C‐37.5°C) by using different mechanisms such as sweating and vasodilation. If the regulatory mechanisms are not able to level off the thermal energy income with the cooling losses, the thermal balance remains impaired, causing a further temperature rise. Every time the body core temperature is outside the thermoneutrality range, cellular physiological processes gradually begin to alter, which can impair organ performance and regulatory function. If this situation is prolonged, the individual may experience heat exhaustion, which may potentially develop into heatstroke, considered a life‐threatening illness. This scenario is quite rare for young adults but more likely to happen in the case of aged people. This is due to the fact that thermal resistance can be much lower at old age, and therefore, such processes may occur much faster, without allowing the subject to take adequate countermeasures. Theoretical evaluation of the temperature distribution in an aged body under thermal stress may therefore provide valuable insights on ways to prevent the onset of such pathological conditions. Furthermore, modelling heat transfer in an aged human body may also be useful for other applications, such as temperature‐controlled surgeries, and age‐related diseases, such as Alzheimer's.

To study heat transport in the human body, several different bioheat transfer models^27^, ^28^, ^29^, ^30^, ^31^, ^32^, ^33^, ^34^, ^35^ were proposed in the recent past, ranging from simple lumped models to more complex realistic 3D representations of the body. Although most of these works introduce comprehensive modelling methodologies, ageing effects on the body's thermal energy balance are not generally taken into account. To the best of the author's knowledge, a limited number of works were carried out on the topic of ageing effects on human body thermal energy balance. A significant contribution is made by the work of Novieto, 24 which models ageing effects on the body by modifying parameters such as metabolic rate, CO, body weight, and height and body surface area. Age model modifications were made according to experimental observations.^25^, ^36^, ^37^, ^38^, ^39^ To account for ageing, Rida et al^40^ modified the regulated blood perfusion coefficients at the skin, the metabolic rate during hot/cold conditions, and decreased the CO. Predictions showed a good agreement with the experimental results, for both cold and hot thermal stress situations. Hirata et al^41^ attempted to evaluate the core temperature, average skin temperature, and skin mass evaporation for a body subjected to hot external conditions (>40°C). In this work, no significant age variations in CO and tissue volumes were assumed, but sweating losses depending on age were included. Predicted results agreed with the experimental data obtained for 2 age categories. With regard to the ageing effects on the arterial blood flow, relevant contributions were made by the works of Maksuti et al^42^ and Pagoulatou and Stergiopulos.^43^ In these works, arteries are subjected to a progressive stiffening with advancing age, leading to a higher flow resistance in the bloodstream. The resulting pressure overload is compensated by a left ventricle (LV) thickening, with a consequent alteration of the cardiac function. Modelling of these components was validated against experimental data and the global methodology was shown to achieve excellent agreement between simulations and flow measurements. Another relevant study was carried out by Guala et al,^44^ which proposed a modelling strategy describing aortic stiffening and remodelling compensation during ageing. Zulliger and Stergiopulos^45^ provided a valuable contribution on vascular ageing, by proposing a new strain energy function accounting for ageing in the human aorta.

It appears that a small number of conduction‐based ageing models have been used to study the body thermal energy balance. It is also obvious that some attempts have been made to quantify the effect of ageing on blood flow. However, a comprehensive model that combines the effect of blood flow, heat convection, and bioheat transfer to study the ageing‐induced changes is not available. Thus, in the present work, a comprehensive thermal energy balance model that incorporates all major features mentioned above is evaluated, allowing to identify the components that are strongly relevant in modelling heat transfer in an ageing human body. It is important to note that the proposed methodology is based on the bioheat transfer framework introduced by Coccarelli et al.^33^ In Section 2, the most relevant changes due to ageing are presented, and some numerical examples are discussed in Section 3. These results show the impact of changes due to ageing on the body thermal energy balance (represented by means of body thermal indicators, such as *T*_*core*_ and average skin temperature ${\bar{T}}_{sk}$). This is then followed by a final section where concluding remarks are derived.

## 2 MODELLING THE EFFECTS OF AGEING ON THE HUMAN BODY

In the following subsections, a comprehensive, age‐dependent modelling methodology describing heat transfer within a human body is presented. The whole system can be subdivided into 3 major subcomponents: the arterial systemic circulation, the solid tissues, which constitute the passive system, and the thermoregulatory system. For each model subcomponent, the formulation/parameter dependencies on age are highlighted.

### 2.1 Arterial system

The arterial system is represented by a network of elastic tubes, with the LV as the inlet of the circuit and peripheral circulation as terminals. Some relevant contributions on 1D blood flow modelling can be found in recent studies.^46^, ^47^, ^48^, ^49^, ^50^, ^51^, ^52^, ^53^, ^54^, ^55^, ^56^, ^57^

#### 2.1.1 1D flow in elastic tubes

The variables considered for describing blood flow in 1D elastic vessels are the cross‐sectional area (*A*), the cross‐sectionally averaged velocity (*u*), and the average temperature (*T*) in a cross section. The flow is assumed to be laminar, incompressible (density *ρ* constant) and Newtonian (viscosity *μ* constant). Pressure (*p*) is related to area *A* via the classical non‐linear relationship,^58^, ^59^ ie,

(1) $$p=p_{ext}+\beta (\sqrt{A}-\sqrt{A_{0}}),$$

where *p*_*ext*_ is the transmural pressure, *A*_0_ is the unstressed cross‐section area, and *β* is a parameter representing the wall elasticity. The latter parameter can be expressed as

(2) $$\beta =\frac{\sqrt{\pi }h_{w}E}{A_{0}(1-\sigma^{2})},$$

where *h*_*w*_ is the wall thickness, *E* is the Young's modulus whilst *σ* is the Poisson's ratio (assumed to be 0.5 for incompressible vessel walls). For an artery, the variation in wall stiffness with age can be estimated from the changes of the pulse wave velocity.^42^, ^43^ To model arterial stiffening for a Windkessel model, Maksuti et al^42^ decreased the compliance inversely proportional to the pulse wave velocity. The pulse wave velocity can be seen as the intrinsic wave speed (*c*) associated to the vessel, given as

(3) $$c=\sqrt{\frac{\beta \sqrt{A}}{2\rho }}.$$

Because *c* value at the age of 80 is twice to that of the value at 20, the compliance at 80 is decreased by a factor of 4. If such stiffness change is assumed to be uniform along the network, the variations in *c* can be adopted for representing *β* variations. In the work of Pagoulatou and Stergiopulos,^43^ a set of empirical inverse relationships between arterial diameter *d* (in mm) and pulse wave velocity (in m/s) was derived for all ages in intervals of 10 years as

(4) $$c=\frac{ā}{d^{\bar{b}}},$$

where ā and $\bar{b}$ are fitting coefficients depending on the age (see Table 1).

**Table 1:** Age‐dependent fitting coefficients of Equation 4, from the study by Pagoulatou and Stergiopulos^43^

*ξ*, y	ā	$\bar{b}$
30	15.48	0.502
40	15.59	0.458
50	16.33	0.447
60	16.68	0.428
70	15.91	0.372
80	15.29	0.345

By combining Equations 3 and 4, it is possible to calculate *β* for each artery. This was done under the assumption that the lumen diameter does not change with age. Benetos et al^8^ showed that arterial distensibility decreases significantly only in coronary arteries whilst in femoral arteries the variation is modest. From this study, the percentage decrease in distensibility (stiffness doubled from 30 to 60 years) matches well the assumption made by Maksuti et al.^42^ It is noteworthy that uniform stiffening augmentation should mainly be considered for arteries belonging to the body trunk and not localised in the limbs.

Fluid thermal properties such as specific heat (*c*_*p*_) and thermal diffusivity (*α*) are also considered constant. The inner wall heat transfer coefficient *h*_*in*_ is calculated by assuming the Nusselt number equal to 4.^60^ The conservation laws for mass, momentum, and energy can be written in the following compact form:^61^, ^62^, ^63^, ^64^

(5) $$\frac{\partial \bar{U}}{\partial t}+H\frac{\partial \bar{U}}{\partial x}+\frac{\partial \bar{G}}{\partial x}=\bar{S},$$

with

$$\bar{U}=\left[\begin{array}{c} A\\ u\\ T \end{array}\right],\;H=\left[\begin{array}{ccc} u & A & 0\\ \frac{\beta }{2\rho \sqrt{A}} & u & 0\\ 0 & 0 & u \end{array}\right],\;\bar{G}=\left[\begin{array}{c} 0\\ 0\\ -\alpha \frac{\partial T}{\partial x} \end{array}\right]\;\text{and}\;\bar{S}=\left[\begin{array}{c} 0\\ -\frac{8\pi \mu }{\rho }\frac{u}{A}\\ \frac{2h_{in}}{\rho c_{p}\sqrt{A/\pi }}(T_{w}-T) \end{array}\right],$$

where $\bar{U}$, $\bar{G}$, and $\bar{S}$ are, respectively, the primitive variables vector, the diffusive, and source terms, whilst **H** is the Jacobian matrix associated to the system. *T*_*w*_ is the wall temperature and corresponds to the temperature of the tissue node in contact with the fluid. Equation 5 is solved by employing the fully explicit locally conservative Taylor‐Galerkin method. 62, 65, 66, 67, 68

Because system (5) is hyperbolic (for diffusive terms equal to 0) and the flow is subsonic, boundary conditions at the inlet and at the exit are required for each primitive variable. The prescription of inlet and outlet variables is carried out by means of characteristic variables, defined as^33^, ^62^

(6) $$w_{1}=u+4\sqrt{\frac{\beta \sqrt{A}}{2\rho }},\;w_{2}=u-4\sqrt{\frac{\beta \sqrt{A}}{2\rho }}\;\text{and}\;w_{3}=T.$$

These variables are also used for transmitting information in vessel branching, discontinuities, and terminals. At the network inlet, a forward characteristic variable is prescribed, according to the pumping function of the heart (see Section 2.1.3).

#### 2.1.2 Arterial tree

For representing the larger arterial system, the network of vessels proposed by Low et al^69^ is used. Such an arterial tree is composed of 91 segments (28 tapering vessels), 6288 elements, and 6379 nodes. The geometry and structural properties of this vasculature are assumed to represent a young adult body (with a reference age *ξ*_0_= 30 years).

The terminal part of the arterial network, also called microcirculation, represents the site of greatest pressure drop in the arterial blood circuit.^70^ Because the number of arterial branches increases dramatically towards the periphery, it is generally convenient to represent the peripheral circulation effects by imposing specific conditions at the outlet nodes of the large arterial network. Several strategies can be adopted for modelling such boundary conditions. Among these, the Windkessel model and tapering vessels represent popular choices. In the first case, a vascular resistance (*R*_*T*_) and a characteristic impedance (*Z*_*T*_) define the terminal behaviour, whilst in the latter, the terminal resistances can be modulated by varying the area and thus *β* of the taper. In several studies,^71^, ^72^, ^73^ no phase difference between reflected flow and pressure at the terminals was assumed, and the downstream effects on flow were considered purely resistive. Terminal reflections can be described assuming that the change in the outgoing characteristic is determined from the change in the incoming characteristic as^62^

(7) $$w_{2}^{n+1}=w_{2}^{0}-R_{R}(w_{1}^{n+1}-w_{1}^{0}),$$

where *R*_*R*_ is the reflection coefficient, $w_{1}^{0}$ and $w_{2}^{0}$ are the initial values of *w*_1_ and *w*_2_, whilst $w_{1}^{n+1}$ can be extrapolated from the previous time step because

(8) $$w_{1}^{n+1}{|}_{x=x_{L}}=w_{1}^{n}{|}_{x=x_{L}-\lambda_{1}^{n}\Delta t},$$

in which *x*_*L*_ is the outlet coordinate, and *λ*_1_ is a variable related to the characteristic wave speed ( $\lambda_{1}=u+\sqrt{\frac{\beta \sqrt{A}}{2\rho }}$). In the current study, extremities are represented by tapering vessels. These terminal vessels present a step decrease in *A*_0_ (and therefore a step increase in *β*) that allows accounting for the characteristic reflections of the downstream vasculature.

Segers et al^9^ showed that the age‐related decrease in elasticity occurring for larger arteries is not fully paralleled by an increase in arterial impedance. Based on this, Maksuti et al^42^ accounted for such effect by increasing linearly (+5*%* per decade) the terminal resistance *R*_*T*_ from 0.8 mmHg s/mL at 20 years to 1.04 mmHg s/mL at 80 years. This can be done also for the tapering model, by varying *β* of each vessel accordingly. It is important to note that the modulation of terminal resistance may affect significantly the CO. Based on experimental evidence,^36^, ^74^, ^75^ Pagoulatou and Stergioupulos^43^ modelled terminal changes by assuming that the resting CO is age independent. They assumed that the resistance increase is inline with the mean arterial pressure rise. From the above mentioned works, it is possible to extrapolate (via least square method) the expressions reported in Table 2. If tapering vessels are used for representing terminals, the wall elasticity *β* is modified according to *R*_*T*_ age variations.

**Table 2:** Set of equations used for modelling terminal resistance variations

	*ξ*_0_, y	Equation
Terminal coefficient $\frac{R_{T}}{R_{T,0}}$	20	0.7722 + 0.009365*ξ* − 6.149·10^−5^ *ξ* ^2^ (Pagoulatou et al^43^
Terminal coefficient $\frac{R_{T}}{R_{T,0}}$	20	0.9 + 0.005*ξ* (Maksuti et al^42^

Any modifications to the peripheral resistance must be such that CO remains within the defined physiological range of approximately 6 to 7 L/min. The temperature condition at the inlet is straightforward to prescribe and is associated with the core tissue temperature (see Section 2.3). At the exiting nodes, temperature is extrapolated in time if velocity is positive; otherwise, the fluid is assumed to be in thermal equilibrium with the interacting tissue node. Variables at the discontinuities are sought by solving a non‐linear system of equations, accounting for the conservation of mass, momentum, and energy and by extrapolating the characteristic variables for the parent and daughter vessels nodes.^62^, ^63^

#### 2.1.3 Heart model

The pumping action of the heart is defined by using a lumped model, which accounts for both LV dynamics and aortic valve (AV) reflections. The cardiac contractile function is commonly represented by a time‐varying elastance model of the LV.^76^, ^77^ This elastance curve (*E*_*LV*_) is commonly used for linking the left ventricular pressure *p*_*LV*_ to the chamber volume *V*_*LV*_ during a cardiac cycle:

(9) $$p_{LV}=E_{LV}(V_{LV}-V_{LV,0}),$$

where *V*_*LV*,0_ is the unloaded ventricular volume. The elastance curve varies cyclically between values defined as end‐diastolic elastance (*E*_*LV*,*min*_) and end‐systolic elastance (*E*_*LV*,*max*_). Stergiopulos et al^78^ approximated the elastance curve by means of a double Hill function:

(10) $$E_{LV}=E_{LV,min}+E_{LV,max}\psi \left[\frac{{\left(\frac{t}{\zeta_{1}T_{card}}\right)}^{\eta_{1}}}{{\left(1+\frac{t}{\zeta_{1}T_{card}}\right)}^{\eta_{1}}}\frac{1}{{\left(1+\frac{t}{\zeta_{2}T_{card}}\right)}^{\eta_{2}}}\right],$$

where *ψ* is a normalisation parameter, T_*card*_ is the cardiac period, whilst *η*_1_, *η*_2_, *ζ*_1_, *ζ*_2_ are parameters determining, respectively, the steepness of the curves and the relative appearance times.

The LV volume at the beginning of the cardiac cycle depends on the end‐diastolic pressure *p*_*ED*_ and can be calculated as *V*_*LV*,*ED*_  =  *V*_*LV*,0_ + *p*_*ED*_/*E*_*LV*,*min*_. In the first cardiac phase (isovolumetric contraction) the chamber volume remains constant whilst pressure increases, until the aortic valve opens. During the ejection phase, the LV volume *V*_*LV*_ varies in time according to^79^

(11) $$\frac{dV_{LV}}{dt}=-G_{AV},$$

in which *G*_*AV*_ is the net flow at the inlet node (after the AV). Once the elastance function *E*_*LV*_ reaches *E*_*LV*,*max*_, the ejection phase ends. This is followed by the isovolumetric relaxation phase, which lasts until *p*_*LV*_ equalises the end‐diastolic pressure (*p*_*ED*_). At this point, the refilling phase starts, and the volume increase can be computed as follows^78^:

(12) $$\frac{dV_{LV}}{dt}=\frac{p_{ven}-p_{LV}}{R_{mv}},$$

where *p*_*ven*_ and *R*_*mv*_ represent, respectively, the pressure and the flow resistance of the mitral valve during ventricular filling (the latter is assumed to be equal to 0.0125 mmHg s/mL). It is important to note that for both Equations 11 and 12, the solution is sought by employing forward Euler's method. It is important to mention that for modelling, the left ventricular contraction, models by Arts et al^80^ and Bovendeerd et al^81^ represent comprehensive modelling alternatives. The LV pressure *p*_*LV*_ is used for evaluating the inlet forward characteristic variable $w_{1,in}^{n+1}$, defined as

(13) $$w_{1,in}^{n+1}=w_{2}^{0}+4\sqrt{\frac{2}{\rho }}\sqrt{(p_{LV}-p_{ext})+\beta \sqrt{A_{0}}}.$$

Depending on the AV state, the forward characteristic variable generated in the LV can be completely reflected, partially or completely transmitted to the arterial system. This is carried out in the model by varying periodically in time the AV transmission coefficient (*R*_*AV*_) from 0 to 1. The valve regulation scheme yields^62^, ^69^

(14) $$w_{1}^{n+1}=w_{1}^{0}+(1-R_{AV})(w_{1,in}^{n+1}-w_{1}^{0})-R_{AV}(w_{2}^{n+1}-w_{2}^{0}),$$

in which $w_{1}^{n+1}$ can be extrapolated backwards in time, similarly to what is done for the terminal elements (see Equation 8).

In the studies by Maksuti et al^42^ and Pagoulatou and Stergiopulos,^43^ it was shown how to modify the cardiac function in order to account for ageing effects. Maksuti et al 42 assumed that the ventricular wall stress is preserved with advancing age, and thus, *E*_*LV*,*max*_ was increased proportionally to the systolic pressure. It is important to note that the hypertrophy was not related to either diastolic strain or ventricular wall stress. Because the LV stiffening renders ventricular filling slower, affecting the diastolic function, *E*_*LV*,*min*_ was augmented proportionally to the increase in *E*_*LV*,*max*_. The end‐diastolic volume (*V*_*LV*,*ED*_) is assumed unchanged during cardiac remodelling. To maintain a constant *V*_*LV*,*ED*_ with advancing age, the end‐diastolic pressure (*p*_*ED*_) was increased appropriately. According to physiological data,^82^ Pagoulatou and Stergiopulos^43^ modelled heart rate age decrease from 73.3 bpm at 30 years to 67.3 bpm at 80 years. Table 3 summarises all reference data used to model LV coefficients for each decade.

**Table 3:** LV reference data for each age decade[Link]

*ξ*, y	*E*_*LV*,*max*_, mmHg/mL^42^	*E*_*LV*,*min*_, mmHg/mL^42^	*p*_*ED*_, mmHg	HR, bpm^43^
20	1.00	0.02500	4.134	73.3
30	1.03	0.02575	4.410	73.3
40	1.09	0.02725	4.687	72.1
50	1.16	0.02750	4.964	70.9
60	1.24	0.03100	5.240	69.7
70	1.35	0.03375	5.517	68.5
80	1.51	0.03755	5.794	67.3

Abbreviations: HR, heart rate; LV, left ventricle.

Values for *p*_*ED*_ were obtained by readapting the age relationship extrapolated by Maksuti et al^42^ to a reference value 4.5 mmHg at 35 years.

### 2.2 Solid system

Because the arterial architecture within the tissues is extremely complex, a multidimensional approach for modelling heat transfer may seem necessary. However, using blood perfusion in a 1D conduction model represents a good compromise between accuracy and computational efficiency.^83^

#### 2.2.1 1D conduction through tissues

**Figure 1:**
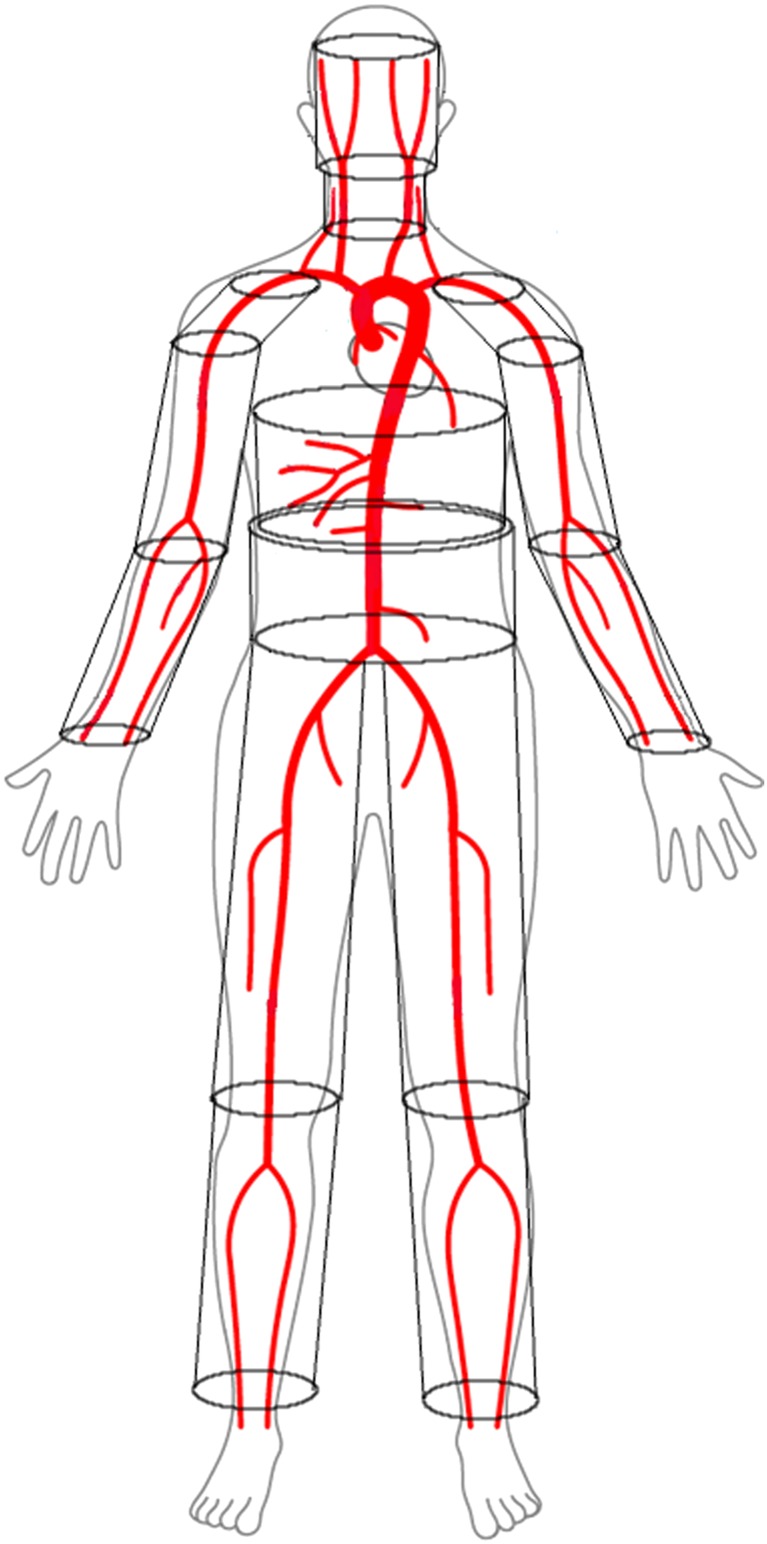
Framework representing the body heat transfer model. Red lines define the larger arterial system, whilst 14 cylinders represent the solid tissues

As shown in Figure 1, the solid system of the body is divided into many solid, circular cylinders. The volume of one of these cylinders is $v_{t}=\pi r_{ext}^{2}l_{s}$ (in which *r*_*ext*_ is the external radius of the cylinder and *l*_*s*_ is the longitudinal length). These cylinders are also divided into layers to describe various forms of tissues and fat (see Section 2.2.2 for more details). The heat conduction in these cylinders is described via the following 1D equation (along the radial coordinate *r*)

(15) $$\rho_{t}c_{t}\frac{\partial T_{t}}{\partial t}-k_{t}\frac{1}{r}\frac{\partial }{\partial r}(r\frac{\partial T_{t}}{\partial r})=q_{v}+c_{p}\rho [\phi (\bar{T}-T_{t})+\frac{2h_{in}}{\rho c_{p}\sqrt{A/\pi }}(T-T_{t})].$$

In the above equation, T_t_ is the tissue temperature; $\bar{T}$ is averaged blood temperature over the tissue section; ρ_t_, c_t_, and k_t_ are, respectively, the density, the specific heat, and the thermal conductivity of the tissue (assumed to be constant within the tissue); and q_v_ represents the volumetric heat production from metabolic activity and is constituted by a basal (q_v,0_) and a variable (q_shiv_) components, whilst ϕ is the blood perfusion coefficient in the tissue. Both q_v_ and ϕ depend on time because they are affected by the thermoregulatory response. The last term of Equation 15 represents the convective source contribution of the vessel lying in the infinitesimal tissue volume (d v_t_  =  π d r ^2^ l_s_). The conduction equation is discretised in space by finite difference and backward Euler's method for the time.^63^

#### 2.2.2 Tissues characterisation and distribution

The solid system is segmented into a set of components/cylinders, each of those characterised by a specific tissues distribution.^33^, ^84^ This consists of 14 cylindrical tissue elements representing head, neck, shoulders, thorax, abdomen, thighs, legs, arms, and forearms (see Figure 1). The segments representing shoulders, legs, thighs, arms, and forearms are constituted by 4 different layers of materials, from inside to outside there are bone, muscle, fat tissues, and skin (see Table 4). For the head, thorax, and abdomen, inner organs are also included (respectively, brain, lung, and viscera). Respiration losses are also included by considering a negative volumetric heat source for all lung nodes.^27^

**Table 4:** Volumic tissue distribution in cylinders from the data of Fiala et al^28^

Cylinder	Tissues	Layer radii, cm	Length, cm
Head	Brain, bone, fat, skin	6.6, 7.6, 7.8, 8.0	23.5
Neck	Bone, muscle, fat, skin	1.9, 5.4, 5.6, 5.8	7.9
Shoulder	Bone, muscle, fat, skin	3.7, 3.9, 4.4, 4.6	13.4
Arm	Bone, muscle, fat, skin	1.5, 3.4, 4.0, 4.2	29.6
Forearm	Bone, muscle, fat, skin	1.5, 3.4, 4.0, 4.2	23.7
Thorax	Lung, bone, muscle, fat, skin	7.7, 8.9, 12.3, 12.6, 12.9	15.6
Abdomen	Viscera, bone, muscle, fat, skin	7.9, 8.3, 10.9, 12.4, 12.6	24.8
Thigh	Bone, muscle, fat, skin	2.2, 4.8, 5.3, 5.5	58.5
Leg	Bone, muscle, fat, skin	2.2, 4.8, 5.3, 5.5	34.3

It is well known that body tissues are subjected to age‐induced modifications. In the study by Kuczmarski et al,^16^ muscle loss was recorded by considering the arm muscle circumference decrease with age. This can be used for approximating the age dependency of the arm muscle thickness (h_mu,arm_). It is also reasonable to assume that the volume reduction rates for all other skeletal muscles, such as the ones in shoulders, forearms, thighs, and legs, are the same. Petrofsky et al^85^ reported, for different age groups, measurements on the fat thickness in different body regions. From their data, the average layer thickness ( ${\bar{h}}_{fat}$) can be linearly described as a function of age. Rida et al^32^ applied a reduction ratio between the fat thickness of young and old adults equal to 0.8. This is inline with the ratio approximately 0.82 (between a 20 and 80 years old) calculated by Petrofsky et al.^85^ In the abdominal region, contrary to the limbs, fat tends to accumulate, and thus, this latter relationship is no longer valid. In the study by Kanehisa et al,^86^ values of abdominal subcutaneous fat and muscle thickness were reported for different age groups (24.2 ± 3.55 and 72.8 ±1.91 years, respectively). With regard to the fat, thickness increases with age from 0.151 up to 0.19 cm, whilst the muscle tissue thickness decreases from 0.139 cm for young to 0.098 cm for elderly. The skin is subjected, with age, to functional and structural changes, which lead to a thinning of the layer. In the study by Petrofsky et al,^85^ the average cutaneous thickness ( ${\bar{h}}_{sk}$) measured for different ages is also reported.

For a more realistic description of the bone density ρ_bo_ reduction with age, the set of measurements on male trochanter bone density reported in the work by Looker et al^87^ can be used. Changes in density also affect properties of the tissue, such as thermal conductivity. Walker et al^88^ extrapolated a quadratic relationship between bone thermal conductivity k_t,bo_ (in W/(m K)) and density, ie,

(16) $$k_{t,bo}=0.0343+0.9935\;\rho_{bo}-0.5305\;\rho_{bo}^{2}.$$

Novieto^24^ reported a weight measurements data set for different age groups,^39^ from which it is possible to calculate the body weight BW (in kg) as a function of age as

(17) $$\text{BW}=51.64+1.328\;\xi -0.01384\;\xi^{2}.$$

From Poehlman et al,^23^ a regression curve describing RMR (in kcal/min) with age can be extrapolated

(18) $$\text{RMR}=1.134+0.008\;\xi -0.00013\;\xi^{2}.$$

The body metabolic volumetric production $\bar{q_{v}}$ (in W/cm^3^) can be estimated by dividing Equation 18 by Equation 17

(19) $$\bar{q_{v}}=\frac{0.06978\;\bar{\rho }(1.134+0.008\;\xi -0.00013\;\xi^{2})}{51.64+1.328\;\xi -0.01384\;\xi^{2}},$$

in which $\bar{\rho }$ is the average density of the body (which can be assumed^89^ constant approximately 1.17 g/cm^3^). It is acceptable to assume that for each body tissue, the metabolic production variation with age is the same, ie, $\frac{\partial (q_{v}/q_{v0})}{\partial \xi }\approx \frac{\partial (\bar{q_{v}}/{\bar{q}}_{v0})}{\partial \xi }$, in which q_v0_ and ${\bar{q}}_{v0}$ are volumetric rates for young adults (ξ_0_  =  20 years). Therefore, the tissue metabolic volumetric heat generation may be calculated with respect to age as

(20) $$\frac{q_{v}}{q_{v,0}}=1.152-0.008897\;\xi +5.596\cdot 10^{-5}\;\xi^{2}.$$

Table 5 summarises the set of equations employed for modelling tissue variations.

**Table 5:** Set of extrapolated equations used for modelling tissue variations

	Body segment	ξ_0_, y	Equation
Muscle thickness $\frac{h_{mu,arm}}{h_{mu,arm,0}}$	Arm, forearm	20	1.070122 − 0.003506ξ (Kuczmarski et al^16^)
Muscle thickness $\frac{h_{mu,abd}}{h_{mu,abd,0}}$	Abdomen	24.2	1.14687 − 0.00607ξ (Kanehisa et al^86^)
Fat thickness $\frac{{\bar{h}}_{fat}}{{\bar{h}}_{fat,0}}$	Any except abdomen	20	1.05938 − 0.002969ξ (Petrofsky et al^85^)
Fat thickness $\frac{h_{fat,abd}}{h_{fat,abd,0}}$	Abdomen	24.2	0.87139 − 0.005314ξ (Kanehisa et al^86^)
Skin thickness $\frac{{\bar{h}}_{sk}}{{\bar{h}}_{sk,0}}$	Any	20	1.01026 − 0.000513ξ (Petrofsky et al^85^)
Bone density $\frac{{\bar{\rho }}_{bo}}{{\bar{\rho }}_{bo,0}}$	Any	20	0.95086 + 0.00443ξ − 6.620. 10^−5^ ξ ^2^ (Looker et al^87^)
Metabolic volumetric rate $\frac{q_{v}}{q_{v,0}}$	Any	20	1.152 − 0.008897ξ + 5.596. 10^−5^ ξ ^2^ (Novieto^24^)

#### 2.2.3 Boundary conditions

The body exchanges heat with the environment through the skin, represented by the outer nodes of each cylinder and by breathing. The flux exchanged between the skin layer, and the outside environment is the sum of the convection losses with the ambient air ( ${\dot{Q}}_{con}$), radiation losses with surrounding surfaces and/or sources ( ${\dot{Q}}_{rad}$), and evaporation of moisture ( ${\dot{Q}}_{swe}$) from the skin. At skin nodes Neumann boundary conditions are imposed as

(21) $$-k_{t}A_{ext}\frac{\partial T_{t}}{\partial r}{|}_{r_{ext}}={\dot{Q}}_{con}+{\dot{Q}}_{rad}+{\dot{Q}}_{swe},$$

where A_ext_  =  2π r_ext_ l_s_. The fluxes ${\dot{Q}}_{con}$ and ${\dot{Q}}_{rad}$ are computed according to Coccarelli et al.^33^ Because sweating is considered as a thermoregulatory mechanism, it is presented in Section 2.3.

Blood‐tissue thermal interaction is modelled as follows. Large arteries are subdivided into different categories (core, central, and transversal), depending on their locations within the solid tissue domain. For each of these, a specific modelling/numerical treatment is applied in order to account for their thermal contributions. Core vessels are assumed to be adiabatic, except for the inlet node, whose temperature is reset at each time step to match the core temperature T_cr_ (see Section 2.3). For the central vessels, convective heat transfer with the tissue takes place at the cylinder's innermost node. Transversal arteries are considered as additional volumetric sources. In the present work, the venous system is not accounted for as the blood velocity in veins is significantly lower than the arterial one, and the back flow is assumed to be in thermal equilibrium with the tissues. An exhaustive explanation on artery‐tissue matching is provided in the work by Coccarelli et al.^33^

### 2.3 Regulatory system

Every time the body core temperature (T_cr_) and averaged skin temperature ( ${\bar{T}}_{sk}$) differ from their thermoneutrality values T_cr,0_ and ${\bar{T}}_{sk,0}$, specific regulatory mechanisms intervene in order to counterbalance the energy impairment. Such a thermoregulatory system is based on a network of thermoreceptors located in several parts of the body and communicating with the hypothalamus, which plays the role of controller. The regulatory mechanisms considered in the current study are sweating, cutaneous vasodilation/constriction, and shivering.

#### 2.3.1 Sweating

Sweating occurs for increasing skin temperature and involves latent heat losses at the external surface of the body. The sweating ratio ${\dot{m}}_{swe}$ for each body segment may also be evaluated by using the formulation proposed by Fiala et al^28^ and reformulated by Hirata et al,^41^ which accounts for ageing effects as

(22) $$\begin{array}{ccc} {\dot{m}}_{swe} & ={\dot{m}}_{swe,0}+\chi \;\theta_{sk}\{[0.8\;tanh\;(0.59\Delta {\bar{T}}_{sk}-0.19)+1.2]\Delta {\bar{T}}_{sk}^{*} & \\ & \;+[5.7\;tanh\;(1.98\Delta T_{cr}-1.03)+6.3]\Delta T_{cr}^{*}\}, \end{array}$$

where ${\dot{m}}_{swe,0}$ is the insensible water loss, θ_sk_ and χ depend on the body region

(23) $$\theta_{sk}=\alpha_{swe}\;2^{\frac{T_{sk}-T_{sk,0}}{10}},$$

in which α_swe_ is a skin distribution coefficient associated to each body segment (see Table 6), whilst

(24) $$\Delta {\bar{T}}_{sk}={\bar{T}}_{sk}-{\bar{T}}_{sk,0}\;\text{and}\;\Delta T_{cr}=T_{cr}-T_{cr,0}.$$

**Table 6:** Body part sweating coefficients^*a*^

	Head	Neck	Thorax	Abdomen	Shoulders	Arm/Forearm	Thigh/Leg
α_swe_	0.149	0.042	0.101	0.181	0.0185	0.0455	0.077
χ	1.0	1.0	1.0	1.0	1.0	1.0	0.6

Such parameters are obtained by readapting the values from the work by Hirata et al^41^ for the current body solid architecture. Head segment includes the face. The coefficients for the arm/forearm and the thigh/leg are obtained by dividing, respectively, the sum of the coefficients for arms and hands, and the sum of coefficients for legs and feet by 4.

The temperature thresholds $\Delta T_{sk}^{*}$ and $\Delta T_{cr}^{*}$ are defined as

(25) $$\begin{array}{ccc} \Delta {\bar{T}}_{sk}^{*} & =\left\{\begin{array}{c} 0\;\;\;\text{if}\;\;\;\Delta {\bar{T}}_{sk}\leq \Delta {\bar{T}}_{sk,dec},\\ \Delta {\bar{T}}_{sk}-\Delta {\bar{T}}_{sk,dec}\;\;\;\text{if}\;\;\;\Delta {\bar{T}}_{sk}>\Delta {\bar{T}}_{sk,dec}, \end{array}\right. & \\ \Delta T_{cr}^{*} & =\left\{\begin{array}{c} 0\;\;\;\text{if}\;\;\;\Delta {\bar{T}}_{cr}\leq \Delta {\bar{T}}_{cr,dec},\\ \Delta {\bar{T}}_{cr}-\Delta {\bar{T}}_{cr,dec}\;\;\;\text{if}\;\;\;\Delta {\bar{T}}_{cr}>\Delta {\bar{T}}_{cr,dec}, \end{array}\right. \end{array}$$

where $\Delta {\bar{T}}_{sk,dec}$ and $\Delta {\bar{T}}_{core,dec}$ are thresholds taking into account the decline of the thermal sensitivity associated with the thermoregulatory signal (see Table 7).

**Table 7:** Decline of the thermal sensitivity $\Delta {\bar{T}}_{sk,dec}$ and $\Delta {\bar{T}}_{cr,dec}$ with age^41^

ξ, y	$\Delta {\bar{T}}_{sk,dec}$, °C	ΔT_cr,dec_, °C
<50	0.0	0.0
>50 and <65	1.5	0.6
>65 and <70	1.5	0.6
>70	1.5	0.4

The skin evaporative losses ${\dot{Q}}_{swe}$ are then evaluated via^41^

(26) $${\dot{Q}}_{swe}=min(\frac{40.6\;{\dot{m}}_{swe}}{A_{sk,glob}},2.2\;h_{swe}f_{cl}(p_{v,sk}-p_{v,a})),$$

in which A_sk,glob_ is the global body skin surface, p_v,sk_ is the water vapour pressure at the skin (normally assumed to be that of saturated water vapour at skin temperature), p_v,a_ is the water pressure in the air, f_cl_ is the clothing area factor (the surface of the clothed body divided by the area of the nude body), and h_swe_ is the evaporative heat transfer coefficient.^90^

#### 2.3.2 Cutaneous vasodilation/vasoconstriction

The mechanisms of vasodilation/constriction regulate the blood perfusion at the skin layer, enhancing/reducing the heat exchanged with the environment. Salloum et al^29^ defined, for each body segment, the blood flow perfusing the skin ${\dot{m}}_{sk}$ (in kg/s), ie,

(27) $${\dot{m}}_{sk}=\frac{{\dot{m}}_{sk,dil}\;{\dot{m}}_{sk,con}}{{\dot{m}}_{sk,0}},$$

where ${\dot{m}}_{sk,0}$, ${\dot{m}}_{sk,dil}$, and ${\dot{m}}_{sk,con}$ are, respectively, the basal, the dilation, and the constriction flow rates. The flow rates ${\dot{m}}_{sk,dil}$ and ${\dot{m}}_{sk,con}$ float between a maximum and minimum values ( ${\dot{m}}_{sk,max}$, ${\dot{m}}_{sk,min}$) as

(28) $$\begin{array}{cc} {\dot{m}}_{sk,dil} & =\left\{\begin{array}{c} {\dot{m}}_{sk,0}\;\;\text{if}\;\;T_{cr}\leq T_{dil,low},\\ \frac{T_{cr}-T_{dil,low}}{T_{dil,upp}-T_{dil,low}}({\dot{m}}_{sk,max}-{\dot{m}}_{sk,0})+{\dot{m}}_{sk,0}\;\;\;\text{if}\;\;\;T_{dil,low}<T_{cr}<T_{dil,upp},\\ {\dot{m}}_{sk,max}\;\;\;\text{if}\;\;\;T_{cr}\geq T_{dil,upp}, \end{array}\right. \end{array}$$

(29) $$\begin{array}{cc} {\dot{m}}_{sk,con} & =\left\{\begin{array}{c} {\dot{m}}_{sk,min}\;\;\;\text{if}\;\;\;{\bar{T}}_{sk}\leq T_{con,low},\\ \frac{{\bar{T}}_{sk}-T_{con,low}}{T_{con,upp}-T_{con,low}}({\dot{m}}_{sk,0}-{\dot{m}}_{sk,min})+{\dot{m}}_{sk,min}\;\;\;\text{if}\;\;\;T_{con,low}<{\bar{T}}_{sk}<T_{con,upp},\\ {\dot{m}}_{sk,0}\;\;\;\text{if}\;\;\;{\bar{T}}_{sk}\geq T_{con,upp}, \end{array}\right. \end{array}$$

in which T_dil,low_, T_dil,upp_, T_con,low_, and T_con,upp_ are the lower and upper threshold temperatures for vessel dilation and constriction (36.8°C, 37.2°C, 27.8°C, and 33.7°C, respectively). The perfusion rate ϕ in Equation 15 can be calculated by dividing the skin blood flow ${\dot{m}}_{sk}$ by the mass of the skin in the segment considered. The flow rates ${\dot{m}}_{sk,0}$, ${\dot{m}}_{sk,max}$, and ${\dot{m}}_{sk,min}$ are reported for each segment in Table 8.

**Table 8:** Body parts cutaneous perfusion coefficients^a^

Body segment	${\dot{m}}_{sk,0}$, c m ^3^/s	${\dot{m}}_{sk,max}$, c m ^3^/s	${\dot{m}}_{sk,min}$, c m ^3^/s
Head/neck	1.681	1.255	4.598
Thorax (chest)/shoulder	0.956	0.0	9.235
Abdomen (pelvis)	0.631	0.0	6.098
Arm	0.253	0.0	2.311
Forearm	0.141	0.0	1.543
Thigh	0.404	0.0	3.459
Leg (calf)	0.181	0.0	2.293

a Such parameters are obtained by readapting the values from the work by Fu et al^91^ for the current body solid architecture. It is important to note that the coefficients for the neck and shoulder are assumed to be equal to the coefficients for the head and thorax, respectively.

During heat exposure, elderly people exhibit a reduction in cutaneous blood perfusion with respect to young adults.^92^ The body temperature threshold for triggering such a mechanism may also be assumed to be lower in aged adults. Similarly, with advancing age, the blood perfusion to the skin during cold conditions does not decrease as much as it does for a young adult. According to Holowats and Kenney,^93^ the cutaneous blood flow attenuation for aged people (60‐90 years) is in the order of 25% to 50% with respect to the one of young adults. In Hirata et al,^41^ such variations do not lead to a significant difference in the global body thermal response. Rida et al^40^ proposed a model accounting for such age‐related variations in vasocutaneous flow perfusion. In this work, a reduction of the lower threshold for maximum vasoconstriction (T_con,low_ assumed to be 10.2°C) led to a better match with the measurements obtained on older adults. Based on experimental evidence,^94^ the elderly threshold for vasoconstriction activation (T_con,upp_) was lowered by 0.5°C representing the delay of thermal signal transmission, whilst the vasodilation thresholds (T_dil,low_, T_dil,upp_), which are controlled by T_cr_, were increased by 0.05°C over the reference value used for younger people. These corrections are made for an aged body (>50 years). The basal skin flow ${\dot{m}}_{sk,0}$ can be considered age independent,^40^ whilst for the maximum and minimum cutaneous flows, the following formulations, derived by linear interpolation of data,^93^ are used:

(30) $${\dot{m}}_{sk,min}=\frac{{\dot{m}}_{sk,min,0}}{\left(1.198-0.007677\;\xi \right)}\;\;\;\text{and}\;\;\;{\dot{m}}_{sk,max}={\dot{m}}_{sk,max,0}(1.198-0.007677\;\xi ),$$

in which ${\dot{m}}_{sk,min,0}$ and ${\dot{m}}_{sk,max,0}$ are the corresponding values for a young adult of 20 years old.

#### 2.3.3 Shivering

Shivering occurs only when T_cr_ is equal or lower than 37.1°C and the shivering threshold T_shiv_ (in °C) can be calculated as^27^

(31) $$T_{shiv}=\left\{\begin{array}{c} 35.5\;\;\;\text{if}\;\;\;T_{cr}\leq 35.8{\;}^{o}C,\\ -10222+570.9\;T_{cr}-7.9455\;T_{cr}^{2}\;\;\;\text{if}\;\;\;35.8{\;}^{o}C\leq T_{cr}\leq 37.1{\;}^{o}C. \end{array}\right.$$

Considering the whole body, the maximum metabolic heat production by shivering Q_shiv,max_ (in W) is calculated as

(32) $$Q_{shiv,max}=\frac{1}{3600}(-1.1861\cdot 10^{9}+6.552\cdot 10^{7}\;T_{cr}-9.0418\cdot 10^{5}\;T_{cr}^{2}).$$

The shivering metabolic heat generation Q_shiv_ depends on the skin temperature and shivering threshold temperature

(33) $$Q_{shiv}=\left\{\begin{array}{c} 0\;\;\;\text{if}\;\;\;T_{sk}\leq (40-T_{shiv}),\\ Q_{shiv,max}\left[1-{\left(\frac{{\bar{T}}_{sk}-20}{T_{shiv}-20}\right)}^{2}\right]\;\text{if}\;(40-T_{shiv})\leq T_{sk}\leq T_{shiv}. \end{array}\right.$$

The shivering volumetric source q_shiv_ (in W/cm^3^) for a single cylinder muscle tissue is calculated as

(34) $$q_{shiv}=\alpha_{shiv}\frac{Q_{shiv}}{V_{mus,glob}},$$

where V_mus,glob_ is the global muscle volume of the body, and α_shiv_ is a coefficient taking into account the muscle volume distribution of each body segment with respect to the total (see Table 9).

**Table 9:** Body parts shivering coefficients^a^

	Head	Neck	Thorax	Abdomen	Shoulder	Arm/forearm	Thigh	Leg
α_shiv_	0.01	0.01	0.257	0.364	0.117	0.0125	0.022	0.016

a These parameters are obtained by readapting the values from the study by Fiala^84^ for the current body solid architecture. Head and shoulders include, respectively, the face and the back.

The shivering production is lower in aged people because the shivering threshold core temperature and muscle mass are lower with respect to young adults. In the work by Sessler,^95^ the shivering threshold of the patients are plotted against the age. For 15 adults younger than 80 years old (58 ± 10 years), the mean of the recorded shivering thresholds was 36.1°C, whilst older people (8 people, 89 ± 7 years) shivered at significantly lower temperature (35.2 ± 0.8°C). The reduction rate obtained by linearly interpolating such values is equal to −0.031°C/y.

## 3 NUMERICAL EXPERIMENTS

In this section, simulated results are shown in order to highlight the impact of the modelling assumptions in relation to the effects of ageing. The numerical calculations are carried out by using a framework based on the bioheat transfer model introduced by Coccarelli et al.^33^

### 3.1 Arterial flow results

**Figure 2:**
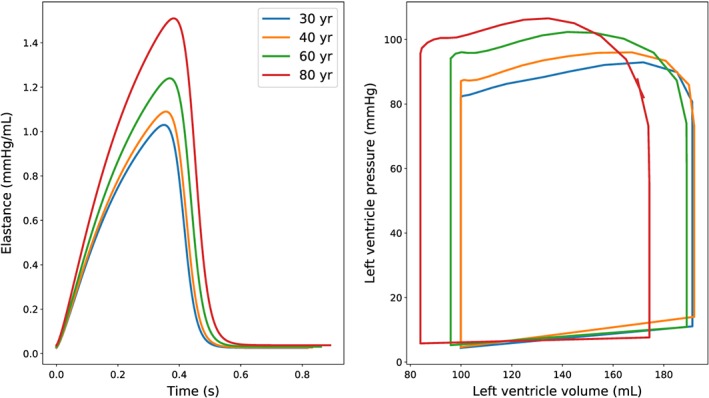
Elastance curve (left) and left ventricle pressure‐volume diagram (right) for different ages. The right plot shows left ventricle quantities calculated immediately after 10 seconds of simulation

In this subsection, the effects of age modifications on flow, presented in Section 2.1, are shown and discussed. Simulations are run considering 4 different ages: 30, 40, 60, and 80 years old. The blood pressure is monitored at 2 sites of the arterial tree: thoracic aorta and brachial artery (corresponding to segments 26 and 29 of the arterial network of Low et al^69^). The time step adopted for these calculations is 2 · 10 ^−5^ seconds. The LV parameters are varied with age according to Table 3. Figure 2 (left) shows how the computed elastance curve varies with age. As seen, the elastance value increases non‐linearly with age. Such variation is also reflected in the PV loop as shown in Figure 2 (right).

The compliance variations in arteries with respect to age are modelled by following the 2 approaches: *uniform* *β* augmentation and *specific* *β* augmentation accounting for pulse wave velocity (through Equation 4). The first strategy is carried out according to the compliance decrease reported by Maksuti et al,^42^ and it is applied to (1) all arteries and (2) only large arteries belonging to the trunk (segments 9, 10, 13, 22, 23, 26, 35, 36, 43, 45, 47, and 49).

It is also worth mentioning that for the uniform *β* augmentation approach, the reference (corresponding to young age) *β* values adopted in the network have a large impact on the simulation results. In the current study, it is assumed that the *β* coefficients derived from the geometrical and material properties proposed by Low et al^69^ are representative of a body at *ξ*_0_  =  40 years. The second strategy is to adapt the vessel pulse wave velocity according to the relationships obtained in the study by Pagoulatou and Stergiopulos.^43^ Terminal resistances (*β* of tapering vessels) are uniformly modulated in order to reflect the terminal resistance increase proposed by Maksuti et al.^42^ The fluid properties employed in the simulations are *ρ*  =  1.06 g/cm^3^, *μ*  =  3.5. 10^−2^ poise, *α*  =  0.0121 cm^2^/s, *c*_*p*_  =  3.9 J/(g °C), and *h*_*in*_  =  0.1 W/(°C cm^2^).

**Figure 3:**
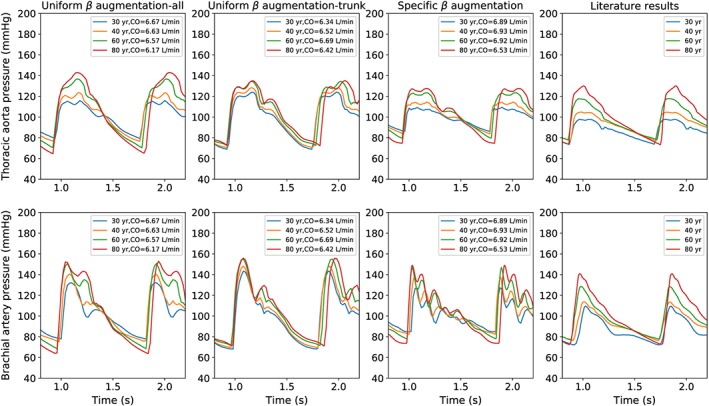
Pressure waveforms at thoracic aorta and brachial artery for different ages (corresponding to the stationary conditions). Literature results are taken from the study by Pagoulatou and Stergiopulos^43^

The analysis of pressure waveforms along the arterial network is of fundamental importance for evaluating the interaction between the cardiac inflow and the vascular resistance. In Figure 3, pressure waveforms at the thoracic aorta and brachial artery are reported for different ages and modelling assumptions. The figure shows that waveform shape is sensitive to the choice of *β* augmentation strategy. In all cases presented, both pressure amplitude and cardiac period are augmented with age. The computed waveforms show a more irregular pattern than the ones presented by Pagoulatou and Stergiopulos.^43^ In the case of specific *β* augmentation, waveforms for young ages (30 and 40 years) are characterised by more than 2 peaks per cardiac cycle. These oscillations may be because Equation 4, underestimating the *β* coefficient for arteries with *d*  <  5 mm,^96^ leads to an extremely compliant downstream vasculature, characterised by an excessively high number of reflections. However, the pressure amplitudes obtained with this approach are the ones that most closely match the reference values.

**Figure 4:**
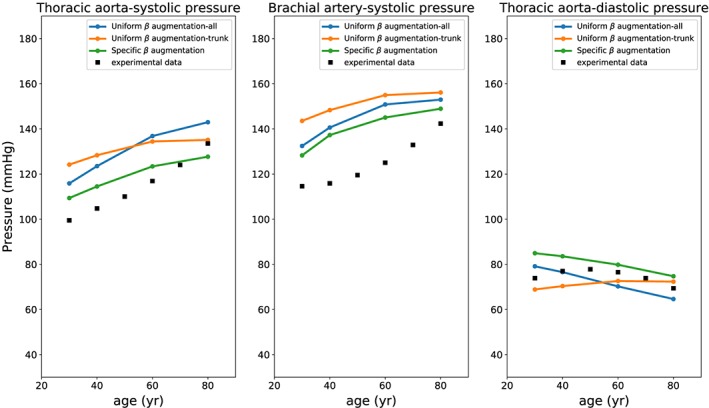
Systolic and diastolic pressure at thoracic aorta and brachial artery for different ages. Experimental data are taken from the studies by Franklin et al^97^ and McEniery et al^98^

Figure 4 shows the age dependency of pressure values against measurements. The simulation results obtained with the specific *β* augmentation method are in good agreement with the experimental observations. The uniform *β* augmentation strategy can be applied for either all arteries, or only core ones, as they do not lead to a substantial differences in the results. However, it is important to say that limiting the age modulation to only trunk arteries leads to better results in terms of diastolic pressures.

**Figure 5:**
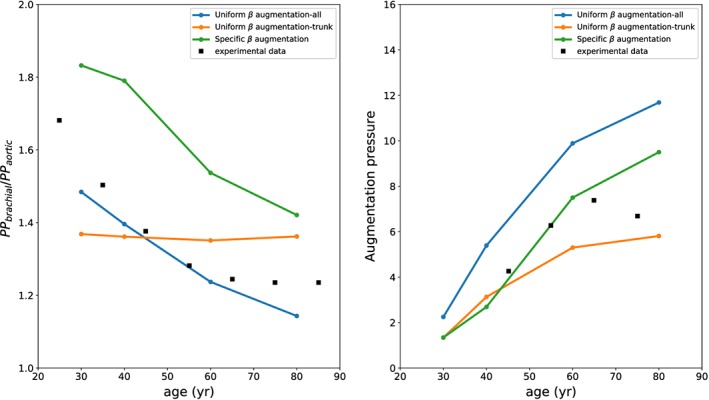
Pressure propagation indicators for different ages. Experimental data are taken from the studies by McEniery et al^98^ (left) and Mitchell et al^99^ (right)

The effects of age‐modelling assumptions are also evaluated by using the ratio between thoracic and aortic pulse pressure and the augmentation pressure (see Figure 5). The employment of such indicators allows to characterise the waveform propagation and reflection. In this case, the specific *β* approach seems to give less accurate results. This may be because, as discussed before, Equation 4 only provides an accurate estimation of pulse wave velocity in large arteries and not for all network vessels.

### 3.2 Tissues heat transfer results

In this section, the effects on thermal balance of age modifications introduced in Sections 2.2 and 2.3 are reported. The flow system variations in vessel compliance are dealt with by adopting the specific *β* augmentation approach. The adopted body‐tissue geometry corresponding to young age (*ξ*_0_  =  30 years) is defined in Table 4. For simulating ageing effects on the tissues, the set of equations reported in Table 5 is employed. The methodologies describing regulatory mechanisms are modified according to Section 2.3. In the case studied here, a bare body is exposed to hot environment (40°C, 42% relative humidity) for 1 hour. The body is assumed to be at resting conditions (no physical activity) and with starting blood and tissue temperatures equal to 37°C. These initial conditions slightly differ from the core and skin temperatures at the beginning of the experimental test. However, as the purpose of the analysis is to evaluate the final thermodynamic state of the body and not to characterise the transients, this discrepancy can be ignored. Simulations are carried out for 2 different ages (30 and 80 years). For these simulations, a time step equal to 10 ^−4^ seconds is employed.

**Figure 6:**
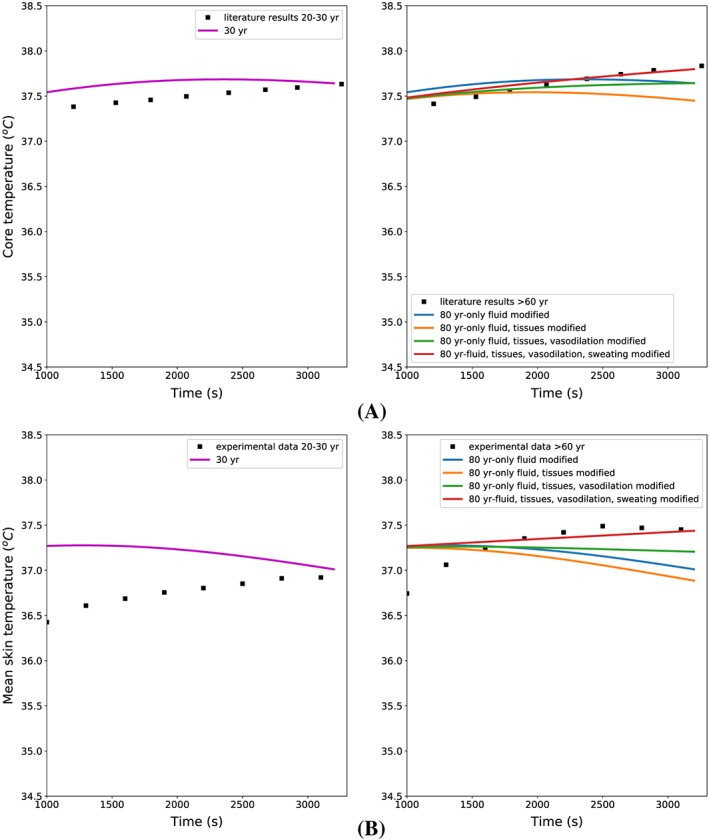
A, Core temperature evolution in time for different modelling assumptions. Literature results are taken from the work by Hirata et al^41^ (the experimental core temperature is assumed to be 37.3°C at the beginning of the test). B, Mean skin temperature evolution in time for different modelling assumptions. Experimental results are taken from the study by Dufour et al^100^ (the experimental skin temperature is assumed to be 34.4°C at the beginning of the test.)

To quantify the body's thermal response, the core temperature and averaged skin temperature are used. Figure 6A shows the core temperature evolution in time for different ages and modelling hypotheses. The case *80 year‐only fluid modified* shows that the age‐related flow changes do not significantly affect the body heat transfer, because the results are the same as the case *30 years*. Tissue property changes, such as volume and metabolic reductions, have a more significant role than arterial convection on the heat balance, as the core temperature drops consistently with time. This occurs because the thermoregulatory system has not been modified and works correctly. A reduced capacity of vasocutaneous perfusion regulation (case where only fluid, tissues, and vasodilation are modified) hinders such a fall in temperature. This effect is further augmented (+0.2°C) if the age deterioration of the sweating mechanism is accounted for. By considering this latter effect, the predicted curve matches better the experimental data. Studying the mean skin temperature evolution in time for different mechanisms activation and age (see Figure 6B) leads to similar conclusions.

**Figure 7:**
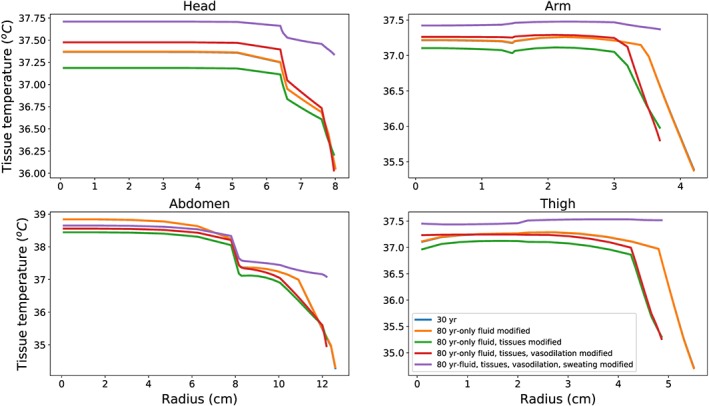
Tissue temperature distributions along radial coordinate at t  =  3000 seconds for different modelling assumptions

In Figure 7, the tissue temperature distributions of head, abdomen, arm, and leg are reported for different modelling hypothesis. These results are inline with what was found previously. The temperatures of the tissues surrounding the arteries are insensitive to age‐induced changes in flow. It is also worth noting that sweating reduction induced by age has a profound effect on the skin temperature. This becomes very evident for the leg section, where the skin temperature becomes higher than the inner tissue.

## 4 CONCLUDING REMARKS

In this work, a comprehensive methodology for modelling age effects on flow and heat transfer in the body is proposed. Each age modification introduced is derived from experimental data or from other computational methodologies. Different approaches for modelling age effects on specific model components are discussed and compared. Computational results obtained for specific age‐associated physiological changes have been tested against literature data. The age modelling strategy for the arterial system is further evaluated by analysing pressure waveforms and other indicators at various sites of the arterial tree. Numerical results show that both *β* augmentation strategies can be employed for representing age‐induced arterial stiffening. With the uniform *β* approach, waveforms are smoother and more similar in shape to the reference ones.^43^ On the other hand, the diastolic and systolic pressures obtained via the specific *β* augmentation strategy are significantly closer to the experimental values.

To elucidate the age dependency of each body thermal component, the body model is exposed to hot stress conditions. This numerical study is characterised by employing body thermal indicators and tissue temperature distributions. Simulation results show that flow variations due to ageing do not have a significant effect on either the global body thermal response or locally on the tissue temperature distribution near the vessel. It is also shown that, under thermal stress conditions, thermoregulatory system deterioration can profoundly affect the body thermal balance. Age‐related dysfunction of sweating and vasodilation mechanisms led to a significant rise in temperature of the internal tissues.

These findings suggest that a limited body capacity to exchange heat with the environment through the skin represents a key factor in the onset of heat‐related pathological conditions. Further detailed research, which incorporates cellular level homeostatic mechanisms in a multidimensional framework, will be required to further understand the effects of ageing on thermal energy balance/imbalance.
